# Emerging therapies for the treatment of rare pediatric bone disorders

**DOI:** 10.3389/fped.2022.1012816

**Published:** 2022-10-11

**Authors:** Kathryn M. Thrailkill, Evangelia Kalaitzoglou, John L. Fowlkes

**Affiliations:** Department of Pediatrics, University of Kentucky Barnstable Brown Diabetes Center, University of Kentucky College of Medicine, Lexington, KY, United States

**Keywords:** achondroplasia, hypophosphatasia, X-linked hypophosphatemia, asfotase alfa, burosumab, vosoritide, fibroblast growth factor-23, C-type natriuretic peptide

## Abstract

In recent years, new therapies for the treatment of rare pediatric bone disorders have emerged, guided by an increasing understanding of the genetic and molecular etiology of these diseases. Herein, we review three such disorders, impacted by debilitating deficits in bone mineralization or cartilage ossification, as well as the novel disease-modifying drugs that are now available to treat these conditions. Specifically, we discuss asfotase alfa, burosumab-twza, and vosoritide, for the treatment of hypophosphatasia, X-linked hypophosphatemia and achondroplasia, respectively. For each skeletal disorder, an overview of the clinical phenotype and natural history of disease is provided, along with a discussion of the clinical pharmacology, mechanism of action and FDA indication for the relevant medication. In each case, a brief review of clinical trial data supporting drug development for each medication is provided. Additionally, guidance as to drug dosing and long-term monitoring of adverse events and pediatric efficacy is presented, to aid the clinician seeking to utilize these novel therapies in their practice, or to become familiar with the healthcare expectations for children receiving these medications through specialized multidisciplinary clinics. The availability of these targeted therapies now significantly augments treatment options for conditions in which past therapy has relied upon less specific, symptomatic medical and orthopedic care.

## Introduction

Monogenetic, pediatric-onset skeletal disorders, while rarer in number, are none-the-less a potential cause of lifelong musculoskeletal abnormalities, physical disabilities, life-impacting comorbidities and often a diminished quality of life. Often times, available therapies have focused on symptomatic and/or supportive care. The identification of disease-specific therapeutic interventions for these unique skeletal disorders has been slower to evolve, in part due to their rarity. However, aided by an understanding of the molecular pathophysiology underlying specific disorders, several novel disease-modifying treatments have been developed in recent years.

In the following pages, we review three such new therapies, specifically developed for the treatment of hypophosphatasia (asfotase alfa), X-linked hypophosphatemia (burosumab-twza), and achondroplasia (vosoritide). These rare skeletal disorders are characterized by defects in bone mineralization, dysregulation of systemic mineral homeostasis, and/or defects in endochondral ossification. Abnormalities in skeletal growth are also a frequent and significant component of disease progression in all three disorders. However, the genetic and molecular abnormalities contributing to each disorder are unique. As a guide for the pediatric clinician, this review provides an overview of each FDA approved drug, its specific mechanism of action and clinical indication, along with clinical guidance for its use and treatment monitoring in pediatric patients (see Table 1 for overview). Current knowledge gaps regarding the safety, efficacy and utility of these therapies are also discussed.

**TABLE 1:** Overview of emerging therapies.

Drug name	Brand name/ Manufacturer/ (FDA approval)	Clinical indication	Pediatric dosing and frequency		Route	Pediatric adverse reactions/ Warnings/ Precautions	Drug interaction(s)
Asfotase alfa	STRENSIQ^®^/ Alexion Pharmaceuticals, Inc.; New Haven, CT (2015)	**Hypophosphatasia** (perinatal/ infantile-, and childhood-onset subtypes)	2 mg/kg, 3× per week, or 1 mg/kg, 6× per week		SQ^1^ injection	ISRs; hypersensitivity; lipodystrophy; ectopic calcifications; possible immunogenicity	Possible interference with laboratory test results
Burosumab-twza	CRYSVITA^®^/ Ultragenyx Pharmaceuticals, Inc.; Novato, CA (2018)	**X-linked Hypophosphatemia** (pediatric and adult, ≥6 months of age)	0.8 mg/kg, rounded to nearest 10 mg; administered every 2 weeks		SQ injection	ISRs; hypersensitivity; cough; GI symptoms; extremity pain/myalgia/spasms; dental disease; hyperphosphatemia/ risk of nephrocalcinosis	Contraindicated with concomitant use of phosphate or Vitamin D analogs, due to risk of hyper-phosphatemia Possible interference with FGF-23 assays^#^
Vosoritide	VOXZOGO™/ BioMarin Pharmaceuticals, Inc.; San Rafael, CA (2021)	**Achondroplasia** (≥5 years of age with open epiphyses)	Weight (kg)	Dose (mg)	SQ injection Once Daily	ISRs; GI symptoms; arthralgia; hypotension (warning); ear pain; influenza; dizziness; fatigue; seasonal allergy; dry skin; potential for immunogenicity	*In vitro* studies show that vosoritide does not inhibit or induce Cytochrome P450 enzymes. No clinical studies evaluating drug interactions of vosoritide have been conducted
			10-11 12-16 17-21 22-32 33-43 44-59 60-89 =90	0.24 0.28 0.32 0.4 0.5 0.6 0.7 0.8			

Therapeutic guidance for Asfotase alfa, Burosumab-twza, and Vosoritide, outlining the clinical indications, dosing information, possible adverse reactions and/or warnings, and possible drug interactions are summarized for each therapy. ^1^SQ, subcutaneous injection; ISR, injection site reactions; GI, gastrointestinal (nausea, vomiting, diarrhea, and constipation). ^#^FGF-23 measurement is not a recommended component of XLH laboratory follow-up in patients on burosumab.

The newer availability of these targeted medications expands the treatment options for conditions in which past therapy has relied upon less specific medical and orthopedic intervention. It is anticipated that use of these medications, where indicated, could contribute to notable improvements in quality of life for individuals affected by these disorders.

## Asfotase alfa

### Clinical pharmacology

#### Drug description

Asfotase alfa (STRENSIQ^®^; Alexion Pharmaceuticals, Inc., New Haven, CT, USA) is a first in class, human recombinant, injectable, tissue non-specific alkaline phosphatase (TNSALP) enzyme replacement therapy, developed specifically for the treatment of pediatric onset hypophosphatasia (1, 2). Its chemical make-up consists of three parts: (1) a 726 amino acid fusion protein incorporating the TNSALP ectodomain (catalytic domain); (2) the human IgG Fc domain, added to prolong circulating half-life of the product (3); and (3) a terminal deca-aspartate peptide, which provides for enhanced targeting of the protein to bone hydroxyapatite (1, 4). Asfotase alfa exists as a soluble glycoprotein composed of two such polypeptide chains, linked by disulfide bonds.

#### Mechanism of action

**FIGURE 1:**
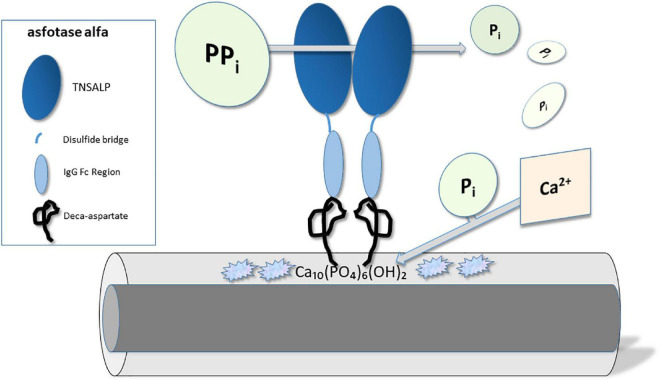
Molecular mechanism of action – Asfotase alfa. Asfotase alfa consists of the tissue non-specific alkaline phosphatase (TNSALP) enzyme linked to the human IgG Fc domain and a terminal deca-aspartate peptide which enhances targeting to hydroxyapatite Ca_10_(PO_4_)_6_(OH)_2_ crystals. TNSALP catalyzes the degradation of pyrophosphate (PPi) into inorganic phosphate (Pi), which in turn can bind calcium, form hydroxyapatite, and improve bone mineralization.

As enzyme replacement therapy for naturally occurring alkaline phosphatase, asfotase alfa functions to replace the specific deficiency in TNSALP existing in persons with hypophosphatasia. By targeting this product to bone hydroxyapatite, alkaline phosphatase (ALP) activity is restored, therapeutically, in proximity to cells involved in bone mineralization. As a result, localized improvements in hydroxyapatite crystal propagation and accumulation (i.e., mineralization) can occur (5) (see Figure 1).

### Targeted disease: Hypophosphatasia

#### Overview

Hypophosphatasia (HPP) is a rare, inheritable, metabolic bone disorder, attributed to loss-of-function mutations in the *ALPL* gene on chromosome 1 (1p36.12), which encodes for the TNSALP. Due to alternative transcriptional and translational processing, various isoforms of TNSALP exist, depending on site of expression (predominantly bones, teeth, liver, and kidney) (3). Pertinent to HPP, TNSALP activities in mineralizing cells of the skeleton (osteoblasts and chondrocytes) and dentition (odontoblasts, ameloblasts, and cementoblasts) are critical to the localized mineralization of these tissues. In contrast, a loss of TNSALP activity results in over-accumulation of three natural substrates for TNSALP, inorganic pyrophosphate (PPi), pyridoxal 5′-phosphate (PLP), and phosphoethanolamine (PEA). PPi, specifically, is a potent inhibitor of hydroxyapatite [HAP; Ca_5_(PO_4_)_3_OH] crystal formation; this defect in HAP crystallization contributes to defective mineralization of bones and teeth, along with disturbances of calcium and phosphorus homeostasis and aberrant calcium crystal deposition in other tissues (6).

To date, >400 genetic variations in *ALPL* (mostly missense mutations, not all being pathogenic) have been identified.^1^ Inheritance of HPP can be autosomal recessive (more common in severe forms of childhood HPP) (7) or autosomal dominant. More specifically, heterozygous missense mutations with a dominant negative effect (DNE) can contribute to both severe HPP, as well as milder forms of HPP showing some residual TNSALP activity. Because homo-dimerization of TNSALP monomers is necessary for enzyme activity, allele-specific protein anomalies capable of disrupting TNSALP dimerization and/or enzyme efficacy can contribute to DNE genetic variants (8). Moreover, this genetic heterogeneity, and the resultant variability in enzyme activity, can explain the broader clinical variability of HPP (detailed below) (8, 9).

#### Clinical features

The hallmark laboratory feature of HPP is a persistently below-normal (age- and sex-adjusted) serum level of alkaline phosphatase. Additionally, an elevated serum level of PLP (circulating form of Vitamin B_6_) is also a sensitive diagnostic biomarker for HPP (2).

The disease is currently classified into six clinical subtypes, based upon the highly variable phenotype presentation, age of onset and severity of disease, as follows (3, 7, 10–12):

(1) Perinatal lethal; a nearly 100% lethal form (13) identified *in utero* or at birth, and characterized by severe bone demineralization (including skull), skeletal deformities, hypoplastic lungs, and critical respiratory compromise.
(2) Perinatal benign; identifiable on prenatal ultrasound, and similarly characterized by poorly mineralized bone, rachitic changes, short and bowed extremities, but with spontaneous late pregnancy or postnatal improvement in the skeletal phenotype contributing to a more varied final outcome.
(3) Infantile; not clinically identifiable at birth, but diagnosed at <6 months of age, and characterized by failure-to-thrive, generalized bone hypomineralization and rickets, hypotonia and muscle weakness. Hypercalcemia, nephrocalcinosis, craniosynostosis and vitamin B_6_-dependent seizures are comorbidities of this form, and a 50% mortality rate is expected, due to respiratory failure (12).
(4) Childhood; mild or severe, diagnosed at ≥6 months to 18 years of age, and characterized by a broad-ranging constellation of symptoms, including rickets, extremity bowing, craniosynostosis, skeletal pain and stiffness, recurrent fractures, muscle weakness and gait abnormalities, short stature, and premature loss of deciduous teeth (a frequent feature).
(5) Adult; diagnosed at ≥18 years of age, but more commonly in middle age, and characterized by osteomalacia, recurrent fractures, musculoskeletal pain, arthropathies and chondrocalcinosis, as well as later-in-life dental abnormalities.
(6) Odontohypophosphatasia (odonto-HPP); characterized by dental features alone (detailed below), but without skeletal manifestations.

Beyond the musculoskeletal features of HPP detailed below, consistent clinical features of the more severe forms of HPP can include respiratory insufficiency or respiratory failure (perinatal/infantile forms), failure to thrive (infantile and childhood forms), hypotonia, vitamin B_6_-dependent seizures, delayed motor milestones, gait abnormalities, hearing loss and fatigue.

#### Musculoskeletal phenotype

Mineralization defects of bones and teeth in HPP can range from severe (perinatal, infantile forms) to mild, correlating with clinical subtype (7, 10). The age-related progression of musculoskeletal features can include severe chest deformities (rachitic chest and gracile ribs), severe skeletal hypomineralization, craniosynostosis, rachitic-like lesions, metaphyseal radiolucencies, hypotonia and musculoskeletal weakness, bowing deformities and limb shortening. With increasing age, propensity to fracture, osteomalacia, milder deficits in bone mineral density, short stature, bone pain, osteoarthropathies, crystal arthropathies, chondrocalcinosis and immobility can result. Dental findings include premature loss of deciduous teeth, lack of cementum and faulty tooth anchoring (i.e., tooth loss with intact roots; tooth mobility), adult tooth loss, abnormal dentin, enamel defects and tooth discoloration, along with excessive dental caries. Biochemical disturbances of mineral homeostasis, more typically seen in severely affected infants and children, can include hypercalcemia, hyperphosphatemia, hypercalciuria, nephrocalcinosis, and ophthalmic calcifications.

#### Prevalence

Disease prevalence is estimated at 1:100,000 in North America and 1:300,000 in Europe (3), but with increased prevalence in select populations such as Manitoba Canadian Mennonites (estimated at 1:2,500) (13).

### FDA approval and indications

STRENSIQ^®^ (Asfotase alfa) injection received initial United States FDA approval in 2015 (14). It is clinically indicated for the treatment of perinatal/infantile-onset and juvenile-onset (childhood) hypophosphatasia. As a recombinant, bone-targeted TNSALP enzyme replacement therapy developed specifically for HPP, it is intended to improve survival, as well as the severe musculoskeletal deficits, functional impairments and metabolic disturbances of the more severe forms of this disease (10).

### Dosage and administration

The STRENSIQ^®^ product is a clear, slightly opalescent, aqueous solution developed for subcutaneous injection as single-dose vials, available in four product concentrations of 18 mg/0.45 mL, 28 mg/0.7 mL, 40 mg/mL, or 80 mg/0.8 mL (80 mg/0.8 mL only for pediatric patients weighing ≥ 40 kg) (14). The recommended dosage for treatment of perinatal/infantile-onset HPP or juvenile-onset HPP is 6 mg/kg per week, administered as either 2 mg/kg, three times per week or 1 mg/kg, six times per week (14) (Dose escalation to 9 mg/kg per week, as 3 mg/kg, three times per week, can be considered for severe perinatal/infantile-onset disease unresponsive to the standard dose). For larger children, total injection volumes of >1 mL require two separate injections at separate sites. Detailed, weight-based and concentration- based dosing guidance is available at https://alexion.com/documents/strensiq_uspi.pdf.

### Adverse reactions

Asfotase alfa is generally well-tolerated, with the most common adverse reactions (ARs) including: injection site reactions (∼60 to 90%) (10, 15, 16); pyrexia (15, 17); lipodystrophy (7–28%; lessened by injection site rotation) (10, 15); musculoskeletal pain (variable sites, ∼10 to 36%) (16, 17); ectopic calcifications (12–14%; including ophthalmologic and renal) (15, 16); and injection-associated hypersensitivity reactions [12%; including rare anaphylaxis (15)]. Additional treatment-emergent but less common adverse events have included craniosynostosis (with increased intracranial pressure), chronic hepatitis, hypocalcemia and/or hungry bone syndrome. For additional details, see https://alexion.com/documents/strensiq_uspi.pdf (14).

### Clinical trial outcomes and skeletal effects

Initial pediatric clinical trials were conducted in infants and young children (≤3 years of age) with perinatal/infantile-onset, life-threatening HPP (1). The first such trial (NCT00744042), a multinational, open-label study of 11 children treated with asfotase alfa for 48 weeks, demonstrated “healing of the skeletal manifestations of hypophosphatasia as well as improved respiratory and motor function” by study end (1). Safety and efficacy data derived from the 7-year, longer-term follow-up of this same study cohort (NCT01205152) demonstrated that the skeletal healing was sustained over time (17). Moreover, improvements in respiratory status, weight gain and growth, gross and fine motor function, and cognitive abilities were reported (17). A second prospective, multinational, open-label study of 69 children (≤5 years of age) with perinatal/infantile-onset HPP treated with asfotase alfa for up to 6 years (NCT01176266) (15) again demonstrated improvements in skeletal manifestations, respiratory function (i.e., ventilator-free survival), linear growth and weight gain. Improvements were evident by 6 months of treatment, and sustained for up to 6 years of treatment. Regarding the primary efficacy measure for this study, HPP-related skeletal disease, “58% of patients were considered responders at month 6 and 72% at Year 1, indicating substantial healing of skeletal manifestations” (15).

Slightly older children (*n* = 13), ages 6–12 years, impaired by either *infantile or childhood* forms of disease, have also been treated with asfotase alfa for 5 years (NCT00952484 and NCT01203826) (18). Data were compared with an untreated historical control group (*n* = 16). Improvements in growth, muscle strength, skeletal mineralization and bone healing were evident by 6 months of therapy, and persisted through 5 years of treatment (18).

More recently, results of a multinational, randomized, open-label study of asfotase alfa therapy in adolescents and adults with any form of HPP (*n* = 19), ages 13–66 years, were published (NCT01163149) (19). This study incorporated a 6-month primary treatment period (randomized 2:1, drug vs. no treatment) followed by a 4.5 year, open-label extension period (all treated). Improvements in ambulation, as evidenced by greater and more independent 6 Minute Walk Test results [6MWT; (20)] were reported. Additionally, some participants experienced greater proximal muscle strength and agility scores, indicating improved functional ability. In contrast, assessments of bone mineralization by transiliac bone biopsy, or of bone mineral density (BMD) by DXA, were not consistently improved by treatment (19). Finally, a retrospective, observational, “real-world” study of adults, aged 19–78 years, but with pediatric-onset HPP (*n* = 14) treated with asfotase alfa for at least 12 months at a single center, documented improvements in physical functioning and health-related quality of life (NCT03418389) (21).

While this review focuses on emerging therapies for pediatric bone disease, a few case reports of off-label use of asfotase alfa in adult-onset HPP have also demonstrated some functional improvements (22) and improved fracture healing (23) as a result of therapy.

### Pediatric monitoring guidelines

In 2017, consensus recommendations of an international expert physician panel, experienced in the care of children with HPP, were published providing healthcare monitoring guidance across the lifespan for HPP patients treated with asfotase alfa (10). Clinicians seeking to treat HPP patients with asfotase alfa would benefit from review of this comprehensive guideline. Additionally, recommendations for monitoring of drug safety are available in this publication (10). We have briefly summarized key (*and commercially available*) components of these recommendations below.

#### Laboratory

Routinely monitored blood tests during asfotase alfa therapy include ALP, plasma PLP, urine PEA, serum calcium (Ca) and phosphate, comprehensive metabolic panel [i.e., electrolytes, BUN, creatinine (Cr), liver function], 25 hydroxy-vitamin D, and urine Ca/Cr, with these tests obtained at baseline, and at 1, 3, 6, or 12 months intervals. Recommended testing frequencies for each test differ by clinical subtype and are detailed by Kishnani et al. (10).

#### Radiographic

A comprehensive skeletal survey is recommended at the time of diagnosis. Thereafter, periodic monitoring of chest, knee and wrist radiographs provide assessments of disease progression and/or treatment efficacy. Changes in BMD can be monitored by DXA, per physician discretion, every 2 years in children (10).

#### Respiratory function

A baseline assessment of respiratory function (upper and/or lower airway) is essential for all children with HPP, with follow-up as clinically indicated by baseline findings or by individual symptoms (10). Periodic monitoring for gastroesophageal reflux and aspiration is also recommended.

#### Growth/nutrition

Documentation and tracking of pediatric auxological data, specifically length/height, weight, BMI and head circumference, is recommended at baseline, every 3 months until age 4 years, and then at 6 month intervals throughout growth years. Baseline and annual nutritional assessment is important for identifying dietary deficits.

#### Physical function (motor milestones, mobility, gait, and muscle strength)

A variety of tests can be used to monitor physical function, including physical therapy/occupational therapy assessments, Bayley Scales of Infant and Toddler Development, 6MWT, the Alberta Infant Motor Scale, the Gross Motor Function Measure, and dynamometer strength testing, along with age-appropriate assessments of gait. The choice of test and testing frequency varies with age (10).

#### Pain and quality of life

Periodic use of age-appropriate pain and quality of life (QOL) assessment questionnaires are recommended, with particular applicability for older children.

### Additional caveats and considerations

The presence of asfotase alfa in blood specimens used for clinical laboratory tests that utilize an ALP enzyme-conjugated immunoassay might result in spurious test results (abnormally high or low) due to drug interference (10, 24, 25). In persons with HPP, use of alternative assays or specimen dilution may be required during asfotase alfa therapy.

### Current research gaps

To date, treatment of individuals with HPP, outside of initial clinical trials, remains of relatively short duration. Moreover, asfotase alfa is currently FDA-approved only for the more severe forms of this disorder, specifically perinatal/infantile-onset and juvenile-onset (childhood) hypophosphatasia. Therefore, only limited data is available as to the durability and tolerability of such treatment over time, and to the impact of asfotase alfa on the natural history of HPP and functional outcomes over time. Additionally, any long-term impact, beneficial or detrimental, on specific organ function (i.e., lung, kidney, eye, other) remains unknown. Finally, the usefulness and/or cost-effectiveness of asfotase alfa for milder forms of HPP has not been delineated. Clinical investigation, along with long-term monitoring, is underway to answer some of these persistent questions. A list of active clinical trials utilizing asfotase alfa can be found at https://clinicaltrials.gov.

## Burosumab

### Clinical pharmacology

#### Drug description

Burosumab-twza (CRYSVITA^®^; Ultragenyx Pharmaceutical, Inc., Novato, CA, USA) is a recombinant human IgG1 monoclonal antibody, directed against fibroblast growth factor (FGF)-23. The product is composed of two heavy chains, each consisting of 447 amino acid residues and two light chain molecules, each consisting of 213 amino acid residues. Each heavy chain has an N-linked carbohydrate moiety at asparagine 297. The product is formulated for subcutaneous injection.

#### Mechanism of action

**FIGURE 2:**
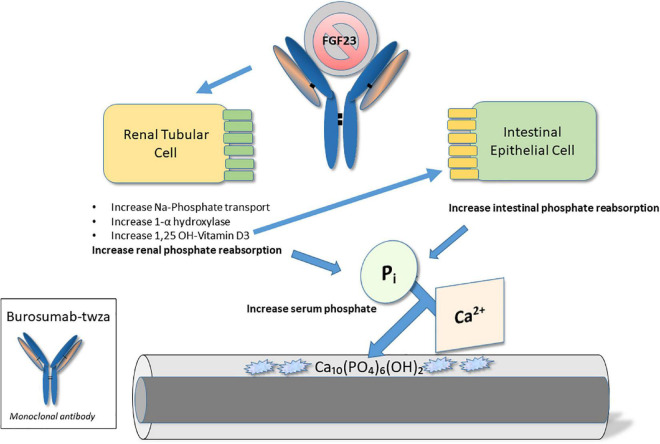
Molecular mechanism of action – Burosumab-twza. Burosumab-twsa is a human IgG1 monoclonal antibody that inhibits fibroblast growth factor (FGF)-23 action in the kidney. In so doing, it directly enhances phosphate (Pi) resorption from the kidney. In addition, through effects on vitamin D metabolism it indirectly enhances Pi reabsorption from the intestine. The resulting increase in serum Pi promotes improved bone quality and mineralization.

Burosumab-twza was developed for the treatment of FGF-23 related hypophosphatemia; however, for the purpose of this review, its indication for the treatment of X-linked hypophosphatemia (XLH) in pediatric patients (but including adult treatment) will be discussed. As detailed below, XLH is characterized by hypophosphatemia, resulting from the impaired reabsorption of inorganic phosphate in renal proximal tubules, due to a systemic excess of the phosphaturic hormone, FGF-23 (26, 27). Burosumab-twza binds to and inhibits the biological activity of FGF-23; as such, the drug is intended to impede FGF-23 signaling in the kidney, thereby restoring renal phosphate reabsorption and normalizing serum phosphate concentrations (28) (see Figure 2).

### Targeted disease: X-linked hypophosphatemia

#### Overview

X-linked hypophosphatemia (XLH) is an inherited form of rickets, resulting from loss-of-function mutations in the gene for PHEX (Phosphate-regulating gene with Homology to Endopeptidases, located on X-chromosome; specifically, Xp22.1) (29), expressed in bones and teeth. Inactivation of PHEX results in an increase in circulating concentrations of osteocyte-derived FGF-23 (30), yet by unknown mechanisms (31). As a major regulator of phosphate homeostasis (26), FGF-23 excess, in turn, contributes to: (1) a *decrease* in renal phosphate reabsorption via down-regulation of Na/Pi co-transport activity in renal tubules; and (2) a *decrease* in synthesis of 1,25(OH)_2_ vitamin D via simultaneous inhibition of 1−α hydroxylase and stimulation of 24-hydroxylase (30, 32). Together, these mechanisms contribute to renal phosphate wasting, limited intestinal phosphate absorption, hypophosphatemia, and resultant abnormalities in mineral deposition in bones and teeth (rickets in growing children; osteomalacia in children and adults; dental disease in all ages) (30).

To date, >300 mutations in PHEX have been identified (30). Inheritance of XLH follows a completely penetrant, X-linked, dominant pattern (27), but with variable severity of phenotype, both within individuals and within kindreds (33). Spontaneous mutations are thought to account for 20–30% of all cases (27, 31).

#### Clinical features

The hallmark laboratory feature of XLH is a persistently below-normal (age-specific) level of serum phosphorus (evident by 3–4 months of age) (27, 34), attributable to decreased renal tubular phosphate reabsorption (low calculated TmP/GFR; tubular maximal reabsorption of phosphate per glomerular filtration rate). Low or inappropriately normal levels of 1,25(OH)_2_ vitamin D are characteristic of XLH; elevated levels of alkaline phosphatase (indicative of rickets) and of FGF-23 can be confirmatory (27, 33). Moreover, in persons without a family history of XLH, genetic analysis of the PHEX gene is diagnostic. Radiographic evidence of rickets (assessed at knees, wrists and/or ankles), including cupped and flared metaphyses, widened and irregular long bone growth plates, and cortical bone thickening, endorses the diagnosis of XLH (34).

Conventional standard-of-care has included daily, multiple-dose oral supplementation with phosphate salts and active vitamin D analogs to rectify hypophosphatemia. However, efficacy of this therapy has been limited by tolerability, adherence, side effects (diarrhea, abdominal pain, and hypercalcemia/hypercalciuria), comorbidities (nephrocalcinosis, hyperparathyroidism, ectopic calcifications, and chronic kidney disease) (27, 35, 36), and requirements for frequent healthcare monitoring.

#### Musculoskeletal phenotype

In children, features of XLH include: rickets (evident beginning ∼6 months of age); lower limb deformities, frequently requiring orthopedic surgical intervention (tibial torsion, lower limb bowing, coxa vera, genu varum, and genu valgum) (33, 34, 37); decreased growth velocity and age-specific short stature; delayed walking and/or gait abnormalities; odontomalacia and dental abscesses (27, 38, 39); craniosynostosis (up to age 5 years) (34, 38, 40); bone and joint pain [specifically, osteomalacia-related bone pain, evident in up to 80% of children (27, 34, 41)]; muscle fatigue and physical dysfunction. Subnormal height Z-scores are evident in children at ≤4 years of age (27, 42).

In adults, the musculoskeletal phenotype primarily reflects the exacerbation of pediatric disease manifestations. Hence, XLH in adults is similarly characterized by lower limb deformities, disproportionate adult short stature, bone pain (41), dental disease and musculoskeletal function deficits (27, 30, 43, 44). However, osteomalacia (present in adults and children) supersedes active rickets (exclusive to growing bone). Consequently, adults with XLH experience frequent fractures and/or pseudo-fractures (27, 45, 46), in addition to weight-bearing osteoarthritis (spine, hips, and knees), enthesopathies (hands, feet, spine, hips, and sacroiliac joints) and hearing loss (30, 33, 43, 47–49). Fractures or pseudo-fractures are reported in 45–60% of adults, and enthesopathies are reported in 100% of patients ≥ 30 years of age (27). Again, orthopedic intervention, including joint replacement, bone stabilization, and spinal surgeries may be indicated (29, 33).

#### Prevalence

X-linked hypophosphatemia is the most common cause of inherited hypophosphatemic rickets. Disease incidence rates of 3.9 per 100,000 live births (34) to 5:100,000 (50) are reported. Disease prevalence has been estimated at between 1.5:100,000 (51) to 1.7:100,000 children (34) and 4.8:100,000 persons (all ages) (34). However, variations in prevalence (from 1:20,000 to 1:60,000) have been reported across different genetic backgrounds (31). XLH accounts for ∼80% of all familial cases of hypophosphatemia (33).

### FDA approval and indications

CRYSVITA^®^ (Burosumab-twza, hereafter referred to as burosumab) received United States FDA approval in April, 2018 (28, 52). It is clinically indicated for the treatment of X-linked hypophosphatemia in adult and pediatric patients ≥ 6 months of age (or ≥1 year of age in Europe) (52, 53). As a monoclonal antibody that binds FGF-23 and inhibits its signaling, it is intended to increase serum phosphorus concentrations by rectifying the underlying defects in renal phosphate reabsorption and 1,25(OH)_2_ vitamin D production. Improvements in rickets severity, growth, physical functioning, pain alleviation, and fracture healing (adult patients) are anticipated in XLH patients in response to therapy. Due to its superiority over conventional therapy, burosumab is now considered first line therapy for symptomatic children with XLH. However, the cost-effectiveness of burosumab vs. conventional therapy in children with a very mild XLH phenotype remains inconclusive (31).

### Dosage and administration

The CRYSVITA^®^ product is a clear, slightly opalescent, and colorless to pale brown-yellow aqueous solution developed for subcutaneous injection as single-dose vials, available in three concentrations of 10, 20, and 30 mg/mL (52). Standard-of-care treatment with oral phosphate and/or vitamin D analogs should be discontinued 1 week prior to starting CRYSVITA; concomitant use of these products is contraindicated during CRYSVITA therapy. For pediatric patients with XLH, the recommended starting dosage is 0.8 mg/kg, rounded to the nearest 10 mg (10–90 mg dose range), and administered every 2 weeks (52). The dose can be increased up to 2 mg/kg (90 mg maximum dose), administered every 2 weeks, if indicated. For adults with XLH (≥18 years of age), a dose of 1 mg/kg, rounded to the nearest 10 mg (90 mg maximum) and administered every 4 weeks is recommended (52). The maximum injection volume for CRYSVITA is 1.5 mL; if multiple injections are required for a single dose, use of separate injection sites is recommended. Follow-up measurement of fasting serum phosphorus in the weeks and months following initiation of therapy is used to guide dose adjustments, with the aim of targeting phosphorus levels to the lower-end of age-appropriate normal ranges. Detailed pediatric and adult dosing schedules for increase or decrease in dose are available at https://www.accessdata.fda.gov/drugsatfda_docs/label/2019/761068s004lbl.pdf.

### Adverse reactions in pediatric patients

Burosumab has been generally well-tolerated by both adult and pediatric patients with XLH (28). Adverse reactions to burosumab therapy, specifically reported in pediatric trials, have included (52): pyrexia; ISRs [∼50 to 58% of pediatric patients (28, 52, 54)]; cough; vomiting/nausea; extremity pain; headache, rash; dental concerns (toothache/caries/tooth abscess); myalgia; diarrhea, and dizziness. Additional treatment-emergent events reported in adult XLH patients have included: nasopharyngitis; back pain; muscle spasms; and restless leg syndrome (28, 52). Hyperphosphatemia was also reported in adults, but managed with CRYSVITA dose reduction.

### Clinical trial outcomes and skeletal effects

Implementing results from phase 1 and 2 studies in adult XLH patients designed to study drug pharmacokinetic/pharmacodynamic properties, mode and frequency of drug delivery, and initial safety/efficacy of burosumab (29, 55, 56), a phase 3, randomized, double-blind, placebo-controlled, multinational study was conducted in 134 adults with XLH, ages 18–65 years (NCT02526160) (43). Participants were treated with burosumab (1 mg/kg) vs. placebo, administered every 4 weeks for 24 weeks, and thereafter all participants received active drug during an extension trial. On average, participants experienced a normalization of serum phosphorus via improved Tmp/GFR and an increase in 1,25 (OH)_2_ vitamin D. Improved fracture healing along with reduction in musculoskeletal stiffness were also noted.

The initial trial in pediatric participants was an open-label, phase 2 study (NCT02163577) of 52 children with XLH, ages 5–12 years, comparing burosumab administered via SQ injection every 2 weeks vs. every 4 weeks (57). Following a 16-week dose escalation period, participants received an additional 48 weeks of treatment. A significant decrease in rickets severity was noted, along with increases in serum phosphorus (better sustained with 2-week dosing) and TmP/GFR. Additionally, improvements in height, physical functioning and pain alleviation were reported. In a second open-label, phase 2 study (NCT02750618) of 13 children, ages 1–4 years, burosumab was administered every 2 weeks, for 64 weeks. Once again, substantial healing of rickets was reported by week 40 of treatment (42).

An open-label, randomized (1:1), phase 3 (NCT02915705) comparison of burosumab vs. standard-of-care therapy (phosphate + Calcitriol) was next completed in 61 children, ages 1–12 years, treated for 64 weeks (58). Significantly greater improvements in rickets severity, limb deformity, serum phosphorus, TmP/GFR, growth and mobility were noted in the burosumab-treated participants at study end. A multi-center, global, 10-year disease monitoring study (NCT03651505) in children and adults with XLH, intended to assess the long-term safety and efficacy of burosumab vs. conventional therapy is ongoing (27, 59).

### Pediatric monitoring guidelines

Multidisciplinary care for children with XLH is often required, involving primary care coordination, along with subspecialty input from endocrinologists, nephrologists, orthopedic surgeons, physical therapists, dentists and genetic counselors, among others (i.e., neurologists, psychologists, otolaryngologists). Alternatively, referral to an XLH center of excellence or a metabolic bone disease clinic could be offered. Below, we have summarized current recommendations for the management and ongoing follow-up of pediatric patients with XLH, particularly as they relate to treatment with burosumab. This brief overview has been assembled by merging guidelines from a 2021 multi-national consensus statement (31, 34), along with published US and manufacturer-specific guidelines (27, 52).

#### Laboratory

Following initiation of burosomab therapy, measurements of *fasting* serum phosphorus and TmP/GFR are recommended at 2, 4, 8, and 12 weeks of initial therapy, as indicative of drug efficacy. Thereafter, laboratory measurement of serum calcium, creatinine, PTH, ALP and *fasting* serum phosphorus, every 1–3 months for children <5 years of age and every 3–6 months for children 5–19 years of age, is recommended. Additionally, biannual 1,25(OH)_2_ vitamin D (safety parameter) and annual 25(OH) vitamin D measurements are recommended. Routine assessment of urine calcium and creatinine should also be obtained at times of other lab testing, to monitor for hypercalciuria (safety parameter). Finally, a follow-up measurement of serum phosphorus is recommended 4 weeks after any burosumab dose adjustment.

#### Radiographic

For follow-up of rachitic disease, knee and wrist radiographs are recommended 6 months after the start of burosumab, and every 1–2 years thereafter (along with a bone age X-ray, as clinically indicated, for children with short stature). Less frequent radiographic evaluation appears justified in those children who are thereafter responding well to therapy and have no other clinical indicators, so as to minimize radiation exposure.

#### Renal function

Routine blood pressure monitoring is recommended for all children. In addition, renal ultrasonography, every 1–2 years is recommended to monitor for the XLH-associated risk of nephrocalcinosis. Ultrasounds should be done annually for patients with nephrocalcinosis or persistent hypercalciuria, and every 2 years in those without prior evidence of these co-morbidities.

#### Growth

Regular pediatric monitoring of anthropometric measurements should occur, including assessment of height, height velocity, weight, BMI, head circumference, and the intercondylar (knee) and intermalleolar (ankle) distances of the lower limbs, as age-appropriate. Physical examination should include craniofacial examination in children <5 years, for evidence of craniosynostosis. A referral for medical nutrition therapy may be indicated in cases of altered BMI.

#### Physical function (motor milestones, mobility, gait, and muscle strength)

Orthopedic referral will be required for symptomatic individuals with XLH-related skeletal deformities. Annual physical therapy assessments are also recommended [with 6MWT (>5 years), timed up-and-go test, gait assessments or other musculoskeletal evaluations, as clinically indicated and/or age-appropriate]. Routine physiotherapy may be required for specific musculoskeletal deficits.

#### Dental

Due to hypomineralization of dental tissues, patients with XLH are at increased risk for dental necrosis, dental abscesses, and periodontitis. Therefore, twice-yearly dental evaluations are recommended in pediatric XLH patients, irrespective of the prescribed therapy.

#### Neurological

Neurologic assessment and/or neurology or neurosurgical referral may be necessary, as indicated by clinical symptoms of headache, papilledema, vomiting, vertigo, neck pain, or other sensory deficits (suggestive of craniosynostosis, Type 1 Chiari malformation, spinal stenosis, etc.). Hearing tests are indicated after age 8 years (34), if hearing impairment is suspected clinically.

#### Pain and quality of life

Age-appropriate annual or biennial assessments of QoL, pain, and depression are recommended.

Overall, after age 5 years, and after 6 months of therapy, medical follow-up at 6-month intervals during pediatric years will accommodate these monitoring recommendations.

### Additional caveats and considerations

Throughout therapy, patients should be advised against the use of any supplemental phosphate or *active* vitamin D analogs while on burosumab. However, if increasing PTH levels are identified during laboratory monitoring, further assessment of nutritional calcium and 25(OH) Vitamin D status is recommended, with supplementation as appropriate (54).

### Current research gaps

Information regarding the long-term safety and efficacy of burosumab in XLH is still unknown; information from an ongoing, 10-year disease monitoring study (NCT03651505), examining the effectiveness of burosumab vs. conventional (or no) therapy on skeletal and renal health should provide this much-needed information. The usefulness and cost-effectiveness of burosumab for children with a milder XLH phenotype has also not been delineated. Finally, the potential efficacy of burosumab in other hypophosphatemic disorders characterized by high circulating concentrations of FGF-23 (i.e., tumor-induced osteomalacia; other forms of hereditary hypophosphatemic rickets; cutaneous skeletal hypophosphatemia syndrome) is under investigation for select conditions. Again, a list of active clinical trials utilizing burosumab can be found at https://clinicaltrials.gov.

## Vosoritide

### Clinical pharmacology

#### Drug description

Vosoritide (VOXZOGO™; BioMarin, San Rafael, CA, USA) is an analog of C-type natriuretic peptide (CNP) developed to increase linear growth in patients with achondroplasia (60, 61). Vosoritide is a peptide consisting of 39 amino acids (proline and glycine on the N-terminus as well as 37 C-terminal amino acids from the natural human CNP) with an extended plasma half-life due to its resistance to neutral-endopeptidase (NEP) digestion (62).

#### Mechanism of action

**FIGURE 3:**
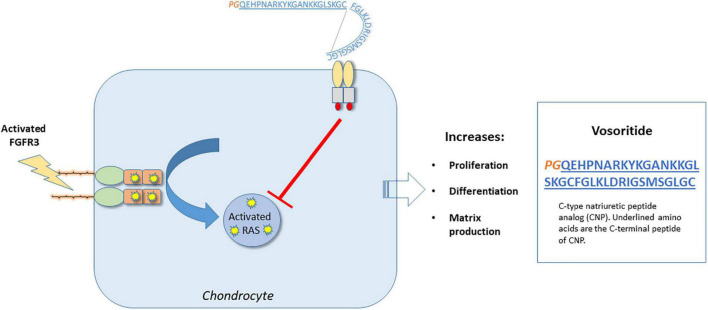
Molecular mechanism of action – Vosoritide. Vosoritide is an analog of C-type natriuretic peptide (CNP), consisting of the last 37 amino acids of the C-terminal peptide of CNP (designated in blue). At the N-terminus, proline and glycine (designated by PG in orange) have been added, which helps to stabilize the peptide. It functions by binding to the C-type natriuretic peptide specific receptor (NPR2) to inhibit FGFR3 signaling at the level of RAF-1. In so doing, it promotes chondrocyte proliferation, differentiation, as well as matrix production.

Vosoritide promotes chondrocyte proliferation and differentiation in the growth plate. Children with achondroplasia have fibroblast growth factor receptor 3 (FGFR3) mutations resulting in constitutive activity of the FGFR3 receptor, resulting in impaired chondrocyte activity and reduced bone growth (63, 64). FGFR3 down-regulates cartilage and bone growth, by inhibiting cell proliferation and differentiation of growth plate chondrocytes (65). Vosoritide binds to the natriuretic peptide receptor type 2 (NPR2), which reduces the activity of FGFR3, by inhibiting the *FGFR3*-mediated mitogen-activated protein kinase (MAPK) signaling pathway (60). By targeting FGFR3 signaling, vosoritide ameliorates the clinical phenotype of achondroplasia (see Figure 3).

### Targeted disease: Achondroplasia

#### Overview

Achondroplasia is the most common skeletal dysplasia in humans (1:15,000–1:30,000 live births), with 80–90% of cases being sporadic (63, 66). The remaining 10–20% of cases are familial due to autosomal dominant transmission (63, 66). The disease is caused by mutations in FGFR3 found on chromosome 4p16.3 (63, 64); FGFR3 is expressed in articular chondrocytes and results in activation of the extracellular signal-related protein kinase (ERK) and p38 of the MAPK pathway (65). As a result, when constitutively active, these signals impair growth-plate chondrocyte proliferation and differentiation, resulting in impaired endochondral ossification (65). Endochondral ossification is responsible for bone elongation; therefore, mutations in the FGFR3 are responsible for the short limbs noted in patients with achondroplasia.

Surprisingly, the majority of the mutations in patients with achondroplasia result from a glycine to arginine substitution at codon 380 (67). Most cases of achondroplasia as noted previously are sporadic and are diagnosed in early infancy or prenatally (66).

### Clinical features

#### Musculoskeletal system and growth

Achondroplasia is characterized by macrocephaly with frontal bossing and a depressed nasal bridge, rhizomelic shortening of limbs, and short stature (66). There is a characteristic “trident” or three-pronged position of the fingers that are short in these patients (66). Patients have thoracolumbar kyphosis and lumbar hyperlordosis (66). Hypermobility of the hips and knees, limited elbow extension, and hypotonia are also present in infants and young children (66). Bowing of the legs often develops in childhood (66).

Individuals with achondroplasia may have normal length at birth; however, they exhibit slow growth and moderate to severe short stature for age (66). Standard growth charts specific for achondroplasia are available and should be used during childhood to assess growth (66). In adult males, average height is 130 cm, whereas in adult females it is 125 cm. The average adult heights are −6 to −7 SD below the mean for unaffected healthy adults (66).

#### Respiratory system

Infants with achondroplasia can have restrictive pulmonary disease, due to a smaller chest, and a small subset of them can develop chronic hypoxemia (66). Although most individuals with achondroplasia will have normal life expectancy, mortality is increased in infants and young children with achondroplasia, likely due to respiratory issues, including compression of vertebral arteries at the cranio-cervical junction and subsequent hypoxic damage of central respiratory control centers in the medulla and restrictive pulmonary disease (66, 68).

#### Other features

Additional medical problems in patients with achondroplasia include: hypertension; gastroesophageal reflux; oromotor hypotonia; failure to thrive; obesity; gross and fine motor delays; language delay; autism spectrum disorders; obstructive sleep apnea; hydrocephalus; increased risk of subdural hematoma; spinal cord damage (cervical myelopathy) due to small foramen magnum; seizures; temporal lobe dysgenesis; middle ear dysfunction; intermittent spinal claudication (neurogenic claudication); lumbosacral spinal stenosis; malocclusion and acanthosis nigricans (66, 68).

#### Prevalence

Disease prevalence is estimated between 0.36 and 0.6 per 10,000 live births in the USA (69) and 3.72 per 100,000 live births in Europe (70). Studies report that older fathers have significantly higher risk of having infants with *de novo* achondroplasia (69, 70).

### FDA approval and indications

VOXZOGO™ (vosoritide) injection received initial United States FDA approval in November of 2021.^2^ Specifically targeted to inhibit downstream signaling of FGFR3, it is intended to increase linear growth in pediatric patients with achondroplasia 5 years of age and older with open epiphyses, indicating growth potential (see text footnote 2).

### Dosage and administration

VOXZOGO is a sterile, white to yellow lyophilized powder that requires reconstitution in sterile water, developed for once-daily subcutaneous injection.^3^ It is available in dosage forms of 0.4, 0.56, or 1.2 mg for reconstitution in a single-dose vial (see text footnote 3). The recommended dosage for treatment is based on patient’s weight (starting at ≥10 kg, see Table 1). It is advised to be given at approximately the same time each day. After reconstitution, VOXZOGO can be held in the vial at a room temperature 20–25°C (68–77°F) for a maximum of 3 h and any residual medication that is not used should be discarded (see text footnote 3).

### Adverse reactions

Vosoritide is well-tolerated, with the most common adverse reactions (ARs) including: injection site reactions (85%); vomiting (27%); arthralgia (15%) decreased blood pressure (13%), ear pain (10%), influenza (10%), gastroenteritis/diarrhea (10–13%), dizziness (10%), fatigue (8%), seasonal allergy (7%), and dry skin (5%) (see text footnote 3) (61).

Specifically, decreased blood pressure was seen in eight out of 60 subjects treated with VOXZOGO (see text footnote 3). The median time to onset from injection was 31 mins with resolution within 5–90 mins. Subjects with significant cardiac or vascular disease and patients on anti-hypertensive medications were excluded from the clinical trials, so it is unclear whether these patients would have higher frequency of hypotensive episodes if treated with vosoritide. There is a warning regarding low blood pressure and instructions for patients to stay well hydrated and have adequate food intake prior to administration to decrease the risk of low blood pressure and associated symptoms. For additional details, see https://www.voxzogo.com/hcp/wp-content/themes/voxzogo-inter-hcp/images/prescribing_information.pdf.

### Clinical trial outcomes and effects on linear growth

Following a phase 2, dose-finding and safety extension study in children ages 5–14 years which showed a mild adverse event profile and sustained increased annual growth velocity after 42 months (NCT02055157 and NCT02724228) (60), clinical outcomes for a phase 3 study were reported. The phase 3 study was a 52-week, randomized, double-blind, multi-center, placebo-controlled trial in children (5 to <18 years of age) with open epiphyses (61). It included 121 subjects from 24 sites in seven countries (Australia, Germany, Japan, Spain, Turkey, USA and UK) (EudraCT number 2015-003836-11; NCT03197766) (61). Participants had a clinical diagnosis of achondroplasia, confirmed genetically, were ambulatory, and had completed at least 6 months of a lead-in observational growth study (NCT01603095) (61). Subsequently, they were randomized to receive vosoritide, 15 μg/kg daily (*n* = 60) or placebo (*n* = 61) (61). Results of this study showed an increase in annual growth velocity of 1.57 cm/year in participants that received vosoritide vs. placebo, as well as a significant difference in change from baseline height Z-score (least-squares mean difference of 0.28, in favor of the vosoritide-treated group) (61). Furthermore, serum collagen type X concentrations were elevated in vosoritide-treated patients compared to patients on placebo. Bone age, dual energy X-ray absorptiometry (DXA) and change from baseline in upper to lower body segment ratio were not different between the treated and placebo groups (61). There were no clinically meaningful differences in change from baseline in health-related quality of life parameters between the two groups (61).

Subsequently, an open-label extension study, where all participants from the previous, phase 3 study were invited to continue on vosoritide (if in vosoritide-treated group, total of 2 years of treatment) or switch from placebo to vosoritide (1 year of treatment), was conducted (NCT03424018) (71). Of the 121 children initially included in the phase 3 study, 119 enrolled in the open-label extension study. Results showed that vosoritide had persistent positive effects on growth, with increases in annualized growth velocity after 2 years in both groups (71). No new adverse effects were reported during this study (71). International expert opinion supports early, long-term treatment to improve adult height and upper to lower segment ratio (72). Expert opinion also suggests that the earlier treatment is started, the more likely it would result in a decrease in symptomatic spinal stenosis, kyphosis and foramen magnum stenosis (72).

### Pediatric monitoring guidelines

Multidisciplinary care for children with achondroplasia is anticipated, including coordinated input from clinical geneticists, developmental specialists and therapists, neurologists and neurosurgeons, pulmonologists, otolaryngologists and orthopedic surgeons, among others. However, specific to the anticipated growth effects of VOXZOGO treatment, the following monitoring guidelines are provided.

#### Growth monitoring

All infants and children should have their body length, weight and head circumference charted in achondroplasia-specific growth charts at every health supervision visit (68). Patients who are on treatment with VOXZOGO should also have their body weight, height and physical development assessed every 3–6 months, with appropriate weight-based adjustments of dose. Discontinuation of treatment should be supported by confirmation of closure of epiphyses.

#### Additional monitoring

All infants and children should have a detailed neurologic evaluation, which might need to include polysomnography and neuroimaging depending on their craniocervical junction risk. Audiology assessment, and orthopedic care for monitoring of the spine are also indicated in patients with achondroplasia (68).

### Current research gaps

Although vosoritide shows promise in improving linear growth in pediatric patients with achondroplasia over the age of five, its use is not US FDA-approved for infants or younger children. In comparison, vosoritide is approved for treatment of patients with achondroplasia aged 2 years and older by the European Medicines Agency.^4^ Future studies are underway to determine its efficacy in younger children (NCT03583697) and to determine whether vosoritide would improve other clinical outcomes in the youngest patients with achondroplasia. Specifically, an ongoing study aims to assess whether vosoritide can prevent or diminish cervicomedullary compression in infants and young children with achondroplasia, as a result of improved growth of the foramen magnum and spinal canal (NCT04554940, EudraCT number, 2020-001055-40) (73). This could potentially reduce the need for future surgery and improve morbidity, mortality, and quality of life for patients with achondroplasia. Additionally, a clinical trial will evaluate vosoritide as a therapeutic agent in selected genetic causes of short stature, such as hypochondroplasia, SHOX deficiency, rasopathies and others (NCT04219007).

## Discussion

Three new therapeutics have been approved recently, for use in specific rare monogenetic pediatric skeletal disorders: hypophosphatasia, X-linked hypophosphatemic rickets and achondroplasia. In each case, drug development has incorporated newer technologies specifically targeting molecular deficits known to be unique to the pathogenesis of each disorder. Moreover, drug development has required well-coordinated, global efforts to identify and recruit substantial numbers of individuals with these uncommon conditions to allow for clinical investigation. In so doing, novel therapeutics have been generated involving enzyme replacement therapy, biologicals, and a drug that functions as a regulator of signaling pathways distal to a disease-specific mutation. The mechanisms of action for these three drugs are depicted in Figures 1–3.

Asfotase alfa was FDA approved in 2015, as a bone-targeted alkaline phosphatase enzyme replacement therapy for the treatment of perinatal/infantile-onset and childhood hypophosphatasia. Drug use in these patient populations has been well-tolerated, and has resulted in sustained healing of the skeletal manifestations of hypophosphatasia, along with improvements in respiratory function, growth, muscle strength and physical functioning.

Burosumab-twza was FDA approved in 2018, as a monoclonal antibody against FGF-23. As a result of FGF-23 signal inhibition, renal phosphate reabsorption and 1,25(OH)_2_ vitamin D production are enhanced, so as to rectify abnormalities of mineral homeostasis and bone mineralization characteristic of X-linked hypophosphatemic rickets. When used for the treatment of *symptomatic* XLH in adult and pediatric patients ≥6 months of age, improvements in rickets severity, growth (pediatric patients), physical functioning, pain alleviation, and fracture healing have been seen.

Vosoritide was FDA approved in 2021, as an analog of C-type natriuretic peptide, capable of binding to the natriuretic peptide receptor type 2 and interrupting downstream signaling of the constitutively active *FGFR3*-mediated MAPK pathway present in achondroplasia. When used in pediatric patients with achondroplasia ≥5 years of age and with open epiphyses, improvements in linear growth have been documented.

For all three disorders, general guidelines for monitoring pediatric patients during these unique drug treatments are provided. However, the authors acknowledge that this review has been written from the perspective of the pediatric endocrinologist, as typically only one component of a multidisciplinary team approach to caring for these patients; consultation with or referral to other specialists is anticipated. Hence, coordination of health care delivery by primary care providers (i.e., a patient-centered medical home model) is likely to optimize the outcomes for these new therapies.

These new therapies now advance the clinical treatment options for rare skeletal disorders, which have lacked targeted medications, but have relied on orthopedic interventions and/or non-specific medical approaches. Use of such creative and targeted methodologies may lead to additional FDA-approved therapies for these conditions as well as other rare disorders of the skeleton for which therapies are lacking. Given the emerging nature of these three treatments, however, longer-term clinical follow-up will be necessary to provide definitive information on the long-term safety and efficacy of these drugs. Additionally, expanded indications for their use will most likely become evident in time.

## Author contributions

KT and EK: writing – review and editing and funding acquisition. JF: writing – review and editing, graphic design, and funding acquisition. All authors contributed to the article and approved the submitted version.

## Footnotes

1
https://alplmutationdatabase.jku.at

2
https://www.fda.gov/news-events/press-announcements/fda-approves-first-drug-improve-growth-children-most-common-form-dwarfism

3
https://www.voxzogo.com/hcp/wp-content/themes/voxzogo-inter-hcp/images/prescribing_information.pdf

4
https://www.ema.europa.eu/en/medicines/human/EPAR/voxzogo
